# Ablation of tau causes an olfactory deficit in a murine model of Parkinson’s disease

**DOI:** 10.1186/s40478-018-0560-y

**Published:** 2018-07-05

**Authors:** Leah C. Beauchamp, Jacky Chan, Lin W. Hung, Benjamin S. Padman, Laura J. Vella, Xiang M. Liu, Bradley Coleman, Ashley I. Bush, Michael Lazarou, Andrew F. Hill, Laura Jacobson, Kevin J. Barnham

**Affiliations:** 1 The Florey Institute of Neuroscience and Mental Health, The University of Melbourne, Parkville, VIC 3010 Australia; 2 The Department of Pharmacology and Therapeutics, The University of Melbourne, Parkville, VIC 3010 Australia; 3 Monash Biomedicine Discovery Institute, Monash University, Clayton, VIC 3800 Australia; 4 The Department of Biochemistry and Molecular Biology, Bio21 Institute, The University of Melbourne, Parkville, VIC 3010 Australia; 5 The Department of Biochemistry and Genetics, La Trobe Institute for Molecular Science, La Trobe University, Bundoora, VIC 3083 Australia

**Keywords:** Tau, Parkinson’s disease, Olfaction, Autophagy, Neurodegeneration

## Abstract

Parkinson’s disease is diagnosed upon the presentation of motor symptoms, resulting from substantial degeneration of dopaminergic neurons in the midbrain. Prior to diagnosis, there is a lengthy prodromal stage in which non-motor symptoms, including olfactory deficits (hyposmia), develop. There is limited information about non-motor impairments and there is a need for directed research into these early pathogenic cellular pathways that precede extensive dopaminergic death in the midbrain. The protein tau has been identified as a genetic risk factor in the development of sporadic PD. Tau knockout mice have been reported as an age-dependent model of PD, and this study has demonstrated that they develop motor deficits at 15-months-old. We have shown that at 7-month-old tau knockout mice present with an overt hyposmic phenotype. This olfactory deficit correlates with an accumulation of α-synuclein, as well as autophagic impairment, in the olfactory bulb. This pathological feature becomes apparent in the striatum and substantia nigra of 15-month-old tau knockout mice, suggesting the potential for a spread of disease. Initial primary cell culture experiments have demonstrated that ablation of tau results in the release of α-synuclein enriched exosomes, providing a potential mechanism for disease spread. These alterations in α-synuclein level as well as a marked autophagy impairment in the tau knockout primary cells recapitulate results seen in the animal model. These data implicate a pathological role for tau in early Parkinson’s disease.

**Electronic supplementary material:**

The online version of this article (10.1186/s40478-018-0560-y) contains supplementary material, which is available to authorized users.

## Introduction

Parkinson’s disease (PD) is a neurodegenerative disease primarily associated with neuronal degeneration of the Substantia Nigra pars compacta (SN). The disease results in severe motor deficits, as the degenerating neurons cannot provide sufficient dopamine, a neurotransmitter that helps control movement, to the axonal terminals located in the striatum. It is established that in addition to the overt motor deficits PD is associated with significant non-motor disturbances, including psychiatric symptoms, dementia, rapid eye movement (REM) sleep behaviour disorder, constipation and a reduced ability to smell odours (hyposmia) [27]. Hyposmia occurs in the prodromal phase of disease and is highly prevalent in both idiopathic and familial PD [34]. Although hyposmia is reported in as many as 90% of idiopathic PD cases [17], little is known about the underlying pathogenesis.

By virtue of its sporadic occurrence, the precise cause(s) of PD is unknown, however a range of genetic and environmental factors that increase the risk of PD developing have been identified. The dominant risk factor that relates to onset of PD is age [54]. The genetic risk factor with the highest population attributable risk (PAR) percent is the *SNCA* gene (12%) encoding the protein α-synuclein (α-syn) [60]. α-syn is the major constituent of Lewy bodies, an evident histopathological feature of PD, and as a result there is a large body of research dedicated to understanding its role in PD. Pathological staging by Braak et al. demonstrates that α-syn accumulation begins in the brainstem and the olfactory system (stage I), before following a topographical spread across the brain, reaching the nigrostriatal system in stage III [10].

The gene with the second highest PAR (8%) is *MAPT*, which encodes for the microtubule-associated protein, tau [60]. Tau has been implicated as a major contributor to disease pathogenesis in a number of neurodegenerative diseases including Alzheimer’s disease where the presence of neurofibrillary tangles (NFTs) consisting of hyper-phosphorylated tau is a defining characteristic. NFTs are also a feature of PD brain, a finding first reported by Lewy himself (reviewed in [24]), and since reproduced a number of times [6, 32]. Despite the genetic and histopathological indications, the role tau plays in PD pathogenesis has not been well characterized.

One of the established biological functions of tau is promotion of the assembly of tubulin into microtubules, thereby stabilising the microtubule structure [18]. Tau has an intrinsically disordered structure that is subject to a number of post-translational modifications (PTMs) [30]. It has been demonstrated that the microtubule assembly role of tau is regulated by phosphorylation, and hyperphosphorylation leads to suppression of this function [3, 43]. It has been hypothesized that abnormal PTMs are a mechanism by which the function of tau is altered, resulting in loss of function, potentially via microtubule destabilisation [61].

The first line of tau^−/−^ mice were generated by Harada et al. and although these animals were shown to have defective microtubule stability and organisation, they were viable and appeared macroscopically normal [25]. However, it has since been demonstrated that behavioural and motor impairments developed in these animals in an age-dependent manner. Lei et al. performed extensive behavioural and neurological investigations into tau^−/−^ animals and showed that these mice display multiple features congruent with PD including an age-dependent motor and cognitive phenotype, iron accumulation and dopaminergic neurodegeneration of the SN [41, 42]. The findings in this study suggest dysfunction of tau is a key pathological event that eventuates in the hallmark pathological feature reported in the PD brain.

The appearance of motor symptoms associated with PD is reflective of advanced disease, as 50–70% of the dopaminergic neurons in the SN have perished by this stage [15, 38]. The advanced disease state currently aligned with diagnosis is a hindrance to the development of neuroprotective drugs and there is a need to develop methods to detect and diagnose patients much earlier in the prodromal phase of disease. As hyposmia is among the first symptoms to appear at the beginning of the prodromal phase, it follows that neuropathology in the olfactory system is an important feature of disease.

Studying olfactory deficits in animal models of PD is valuable in enhancing the understanding of the various mechanisms that may be contributing to PD-related hyposmia. Many animal models have been tested for a hyposmic phenotype [63, 65], and mice overexpressing human α-syn have demonstrated an age-dependent odour detection deficit [73]. Due to the age-dependent nature of the behavioural phenotype in the tau^−/−^ mice, we sought to determine if hyposmia, an early process in the pathogenic pathway of PD, is associated with loss of tau function, using the tau^−/−^ mice as a model.

## Methods

### Mice

Animals were housed according to standard animal care protocols. Rodent chow and water was available ad libitum. Mice were kept on a 12:12 h light dark cycle and all testing was performed during the light phase of the circadian cycle. Sv129B/6 tau^−/−^ mice were bred in house. Wild type (WT) littermate controls (Sv129B/6 tau^+/+^) were used in this study. All studies were conducted in a blinded fashion. All methods conformed to the Australian National Health and Medical Research Council published code of practice for animal research and all experimentation was approved by The Florey Animal Ethics Committee (AEC number: 12–094 and 15–092). Animal numbers (broken down by genotype and sex) are provided in Additional file 1: Table S1*.* Animals were genotyped as part of the breeding strategy and confirmation of tau ablation was performed on tissue via Western Blot (Additional file 1: Figure S2).

### Odour detection test

The Odour Detection Test (ODT) was adapted from [53]. Mice were habituated to vehicle canisters in their home cage for 3 days prior to testing (day 1: single vehicle cannisters; day 2: two vehicle cannisters; day 3: two vehicle cannisters). The test (day 4) was comprised of four 5-min trials (1 h inter-trial interval (ITI)) performed in the home cage in which the mice were exposed to two canisters per trial; one vehicle (400 μL, MilliQ water + 0.1% Tween20) and one novel odour of either 0 (vehicle), 1:10^8^, 1:10^6^ or 1:10^4^ dilutions (400 μL, MilliQ water + 0.1% Tween20 + orange essential oil (In Essence, Australia)). Animals were videoed, and videos manually scored (the scorer was blinded to the experimental conditions) and percentage investigation time was calculated based on the equation: (time spent with novel odour/combined time investigating either canister) X 100. An animal was deemed to be ‘smelling’ if their nose was within a 1–2 cm proximity to the either end of the cannister and appeared to be investigating the cannister (neck extended, whiskers forward). Normal mice will spend more time investigating a novel odour; as such this test determines the concentration at which can detect a novel odour by comparing time spent investigating the two canisters.

### Rota rod

Mice were trained for three sessions on the Rota Rod (Panlab, Spain) 24 h prior to testing. Session 1 was set speed (4 rpm for 2 mins), session 2 was set speed (4 rpm for 2 mins) and session 3 was accelerating (4–40 rpm for 2 mins). During training if the mouse fell off the Rota Rod it was placed back on until 2 min had lapsed. On the test day the Rota Rod was set to accelerating mode (4–40 rpm) over a 5 min trial. Mice were allowed three attempts and the average time of latency to fall was recorded.

### Pole test

Mice were placed vertically (nose up) on a pole (45 cm) that was wrapped in self-adhesive bandage (NexCare, Australia) with a squash ball placed on top to prevent animals climbing and sitting on the top of the pole. Two lines were drawn on the pole to identify a segment of set length (22 cm). One day prior to testing animals were habituated to the pole and allowed five consecutive trials. On the day of testing animals were allowed a further five consecutive attempts which were recorded. Time to turn (animal to complete a 180° rotation) and time to complete were recorded. A successful pole test was defined by an animals ability to complete a full 360 degree rotation and descend the pole nose first using their limbs to ‘walk’ (animals failed if they slid or jumped down the pole).

### Tissue preparation

Animals were euthanized with a lethal dose of sodium pentobarbitone (100 mg/kg, Lethobarb, Jurox, Rutherford, NSW, Australia), and transcardially perfused with cold 0.1 M phosphate-buffered saline (PBS) (Sigma-Aldrich, St. Louis, Missouri, MO, USA), pH 7.4.

The left-brain hemisphere was dissected for regions containing the striatum (caudate nucleus and putamen (CPU)), substantia nigra par compacta (SN) and olfactory bulbs (OB). These fractions were homogenized using a probe sonicator (10 s) in radioimmunoprecipitation assay (RIPA) lysis buffer (150 mM NaCl, 1% nonyl phenoxypolyethoxylethanol (NP-40), 0.5% sodium deoxycholate (DOC), 50 mM Tris (pH 8.0) + protease and phosphatase inhibitors (Complete Mini Protease Inhibitor Cocktail & PhosSTOP Phosphatase Inhibitor Cocktail, Roche Diagnostic)) and butylated hydroxytoluene (BHT) (1:5; tissue weight (mg): buffer volume (μL)). Homogenates were then centrifuged at 10,000 *g* for 20 min at 4°C. The clarified supernatant was collected (cell lysate) and total protein concentrations were determined using the bicinchoninic acid (BCA) assay (Pierce; Rockford, USA) according to the manufacturer’s directions and made to 2 μg/μl aliquots and used for Western Blots.

### Western blotting

Samples were mixed with 4X sample buffer (25 M Tris (pH 6.8), 20% SDS, Glycerol, 100 mg Bromo Blue) containing 100 mM dithiothreitol (DTT), boiled for 5 min and centrifuged at 10000 *g* for 5 mins. Protein was electrophoresed at 270 V for 25 min on 4–20% polyacrylamide gels (BioRad; USA) and transferred on to 0.45 μm (pore-size) nitrocellulose membranes (BioRad; USA). Membranes were blocked in tris-buffered saline with 0.05% Tween20 (TBS-T) containing 5% low-fat milk powder (Diploma, Australia) for 1 h at room temperature, incubated with primary antibodies overnight at 4 °C, and incubated with secondary antibodies for 2 h at room temp. All antibodies (BD Biosciences anti- α-syn, catalogue number: 610786, dilution 1:5000; Novus Biological anti-p62/SQSTM1, catalogue number: 3868, dilution 1:2000; Cell Signalling Technology anti-LC3B(D11)-XP, catalogue number: H00008878-MO1, dilution 1:2000) were diluted in TBS-T containing 5% low-fat milk powder. Membranes were washed in TBS-T for 21 min (3 X 7 min) before and after incubation with secondary antibodies. Proteins were detected using enhanced chemiluminescence (ECL) (BioRad) and visualised with the ChemiDoc (BioRad; USA) and analysed via densitometry (ImageLab 5.2.1, BioRad; USA). Cell lysates were normalised to automated total protein measurement via ChemiDoc stain-free detection software.

### Primary cortical neuron preparation

Primary cortical neurons were isolated from embryonic brain cortices harvested from tau^−/−^ and WT pregnant mice at 14 days of gestation. The neurons were plated in T75 flasks at a density of 150,000 cells/cm^2^ in Neurobasal Medium (cat# 21103049; Life Technologies), and supplement with B-27 serum-free supplement (cat# 10889038; Life Technologies), Glutamax supplement (cat# 35050061; Life Technologies), gentamicin (cat# 15710072; Life Technologies) and incubated at (humidified, 37 °C, 5% CO_2_) for 6 days.

### Exosome isolation

Exosomes were isolated from cell culture media by differential ultracentrifugation. Culture supernatants were collected, and cellular debris was removed by centrifugation at 2000 *g* for 10 min. The supernatant was then centrifuged at 10,000 *g*, for 30 min at 4 °C. The supernatant was collected and centrifuged 100,000 *g* for 1 h at 4 °C to pellet exosomes. The supernatant was discarded and exosome containing pellets resuspended in filtered PBS and re-centrifuged at 100,000 *g* for 1 h at 4 °C to pellet exosomes. The pellet was resuspended in 50 μl PBS.

### Electron microscopy

Exosomes were fixed with 2% glutaraldehyde/PBS, for 30 min at room temperature. 6 μl was applied to a glow-discharged 200 mesh copper grid coated with carbon-Formvar film (ProSciTech, QLD, Australia) and allowed to absorb for 5 min. Grids were washed 2 × with milliQ water and contrasted with 1.5% uranyl acetate. Transmission electron microscopy (TEM) was performed on a Tecnai G^2^ F30 (FEI, Eindhoven, NL) transmission electron microscope operating at 300 kV (Bio21 Molecular Science and Biotechnology Institute, Parkville, VIC, Australia) across 15,000× − 36,000× magnification. Electron micrographs were captured with a Gatan UltraScan® 1000 2 k × 2 k CCD camera (Gatan, Inc., Pleasanton, CA, USA).

### Immunofluorescence confocal microscopy

Cells were cultured on Laminin/Poly-D-Lysine coated glass coverslips for 9 days before experimental treatment. Samples were fixed with 4% (*w*/*v*) paraformaldehyde (PFA) in 0.1 M phosphate buffer (15 min), rinsed three times with PBS, permeabilized with 0.1% (*v*/v) Triton X-100 in PBS (10 min) then blocked with 3% (v/v) goat serum in 0.1% (v/v) Triton X-100/PBS (10 min). The samples were incubated with a rabbit monoclonal LC3B antibody (Cell Signaling Technologies) diluted in 3% (v/v) goat serum in 0.1% (v/v) Triton X-100/PBS for 2 h at room temperature, rinsed three times with PBS, then incubated at room temperature for 2 h with secondary antibodies conjugated to Alexa-Fluor-555 (ThermoFisher). The coverslips were then incubated 0.1% (v/v) Triton X-100/PBS containing 1 μM Hoechst 33,342 (ThermoFisher) and 1 μM DiO (Sigma Aldrich) for 15 min to visualise the cells independently of LC3B staining. The samples were three times with PBS, then mounted using a TRIS buffered DABCO-glycerol mounting medium. All samples were imaged in 3D by optical sectioning using an inverted Leica SP8 confocal laser scanning microscope equipped with an 63×/1.40NA objective (Oil immersion, HC PLAPO, CS2; Leica microsystems), using a z-stack range of 4.8 μm and a voxel size of 180 nm laterally (x,y) and 300 nm axially (z). All figure images were acquired at ambient room temperature using a Leica HyD Hybrid Detector (Leica Microsystems) and the Leica Application Suite X (LASX v2.0.1). All images are displayed as z-stack maximum projections.

### Confocal image analysis

3D image data were processed and analysed using automated image segmentation in Imaris (V8.0; Bitplane). Autophagic vacuole volume was normalized to the cellular volume rather than number of nuclei, as the number of nuclei did not provide a robust measure of cells per image. For volumetric quantification of LC3B positive autophagic vacuoles, images were segmented using 1 μm diameter background subtraction, 150 nm surface smoothing, a manual intensity threshold of 12 (arbitrary), and seeded region growing segmentation (1 μm diameter seed). Cellular volume was quantified by segmentation of DiO staining, using 360 nm surface smoothing and a manual intensity threshold of 10 (arbitrary). Segmented structures smaller than 10 voxels were excluded to remove shot noise.

### Statistical analysis

All data are presented as the means ± S.E.M., and statistical differences evaluated by Student’s *t* test for two sample testing. Odour detection tests were evaluated using a Two-way ANOVA test with repeated measures (one factor repetition) with Fisher LSD post-hoc comparisons for multiple testing, details of ANOVAs and testing of parametric assumptions (Additional file 1: Table S2). For all analyses, *p* < 0.05 was considered to be statistically significant.

## Results

### 7-month-old tau^−/−^ mice have an olfactory deficit that is accompanied by autophagic impairment and accumulation of α-synuclein in the olfactory bulb

**Fig. 1:**
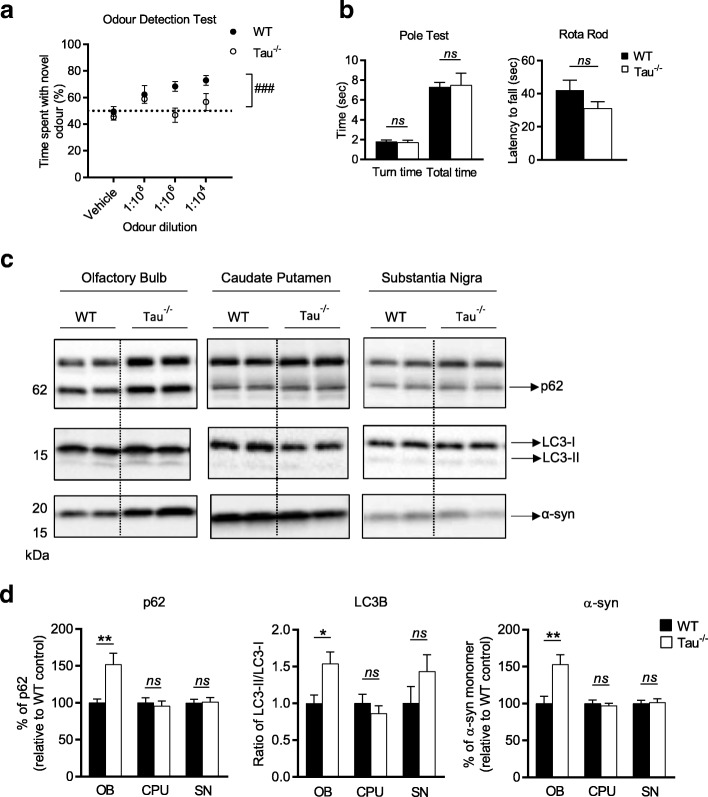
Olfactory deficit, motor performance and pathological features in 7-month-old tau^−/−^ mice. **a** Odour detection test performed on 7-month-old tau^−/−^ (*n* = 10–11) and littermate WT controls (*n* = 10–12). **b** Motor evaluation of 7-month-old tau^−/−^ (*n* = 10) and littermate WT control (*n* = 11), including Rota Rod and Pole Test performance. **c** Representative western blots of cell lysate from olfactory bulb, caudate putamen and substantia nigra from 7-month-old tau^−/−^ (*n* = 6) and littermate WT controls (*n* = 6) immunoblotted for p62, LC3B and α-syn. **d** Quantification of western blot densitometry presented as % of p62 relative to WT control, ratio of LC3-II/I relative to WT control and % of α-syn relative to WT control. Cell lysate for western blots normalised to automated total protein measurement via ChemiDoc stain-free detection software*.* ODT analysed by two-way repeated measures ANOVA (one factor repetition) with Fisher LSD post-hoc comparisons. # represents significant main effect of genotype, ### *p* < 0.001. Motor tests analysed by unpaired two-sided t test. Western blot analysed by unpaired two-sided t test from 3 independent repeats, * *p* < 0.05, ** *p* < 0.01, *** *p* < 0.001, **** *p* < 0.0001, *ns*: not significant. Full western blot images presented in Additional file 1: Figure S3

In order to test whether a loss of tau can impair olfactory capability, 7-month-old tau^−/−^ mice and their WT littermates underwent an ODT (Fig. 1a). This test showed that 7-month-old tau^−/−^ mice showed no preference for investigating the canister containing the novel odour, unlike their WT littermates who showed a significant preference for the novel odour (main effect of genotype *p* = 0.003). At the strongest odour concentration (1:10^4^), a one sample t test with a hypothetical mean of 50% (chance) demonstrated that WT animals were performing significantly above chance, whereas tau^−/−^ animals were not (WT *p* = 0.0001, tau^−/−^*p* = 0.32) (Additional file 1: Table S3). The inability of tau^−/−^ mice to differentiate between a novel odour and a vehicle canister is evidence of a hyposmic phenotype in these animals. In PD, hyposmia occurs prior to the onset of motor deficits. In order to ascertain whether the hyposmia observed in these mice similarly occurs prior to the onset of motor deficits that have previously been reported for these animals, the 7-month-old tau^−/−^ mice underwent motor testing (Fig. 1b). At 7-months-old no difference in motor function was observed between tau^−/−^ and WT mice on either the Rota Rod (*p* = 0.15) or Pole Test (time to turn *p* = 0.76; time to complete *p* = 0.88).

Accumulation and aggregation of protein is a characteristic feature of neurodegenerative diseases and may be the result of impaired clearance mechanisms in affected neurons. Macroautophagy, a major pathway for degradation and recycling of cellular material has been reported to be impaired in PD [45] and implicated in the accumulation of α-syn that is a characteristic feature of the disease [21, 22, 35, 40]. Microtubules, which are reported to be stabilised by tau, play a key role in the endo-lysosomal trafficking process required for autophagic degradation [13, 69]. Autophagosome lysosome fusion is mediated by a number of factors including SNARE Stx17, the HOPS complex and mammalian Atg8 family proteins [29, 31, 33, 49, 50]. Atg8 family members are conjugated to phosphatidylethanoamine on autophagosomal membrane during autophagosome biogenesis, and degraded upon lysosomal fusion with the autophagosome [62]. When analysed by SDS-PAGE, the lapidated form of the LC3B (LC3-II) migrates faster than the cleaved precursor form (LC3-I). As such, an increase in the ratio of LC3-II/LC3-I is often used as a marker of compromised autophagy-mediated degradation [36].

We therefore used LC3B levels to monitor autophagy in tau^−/−^ OB tissue and determine if ablation of tau resulted in alterations to autophagy. A significant increase in LC3-II/I ratio (*p* = 0.01) was detected in the OB of 7-month old tau^−/−^ mice compared to WT mice (Fig. 1d). This increase in the ratio of LC3-II/I was accompanied by a significant 50% increase in the level of p62 (an autophagy receptor that is basally turned over via autophagy) in tau^−/−^ mice (*p* = 0.003) (Fig. 1d) further suggesting 7 mo tau^−/−^ animals have an impairment in the autophagy pathway.

Hyposmia as seen in the human condition has been correlated with an accumulation of aggregated α-syn within Lewy bodies, as demonstrated in Braak staging [10]. Given the suggested autophagy impairment in the tau^−/−^ mice it was of interest to probe the OB for protein accumulation.

Consistent with a PD-like phenotype there was an increase of α-syn in the OB at 7-months-old (*p* = 0.03) (Fig. 1d).

The midbrain (CPU and SN) of these animals was also analysed for LC3B, p62 and α-syn. In line with there being no overt motor deficit, there were no detectable differences in the aforementioned protein levels evident in these brain regions (Fig. 1d). CPU: p62 *p* = 0.65, LC3-II/I *p* = 0.40, α-syn *p* = 0.60; SN: p62 *p* = 0.89, LC3-II/I *p* = 0.19, α-syn *p* = 0.83.

### 15-month-old tau^−/−^ mice have a motor deficit that is accompanied by autophagic impairment and accumulation of α-synuclein in the midbrain

**Fig. 2:**
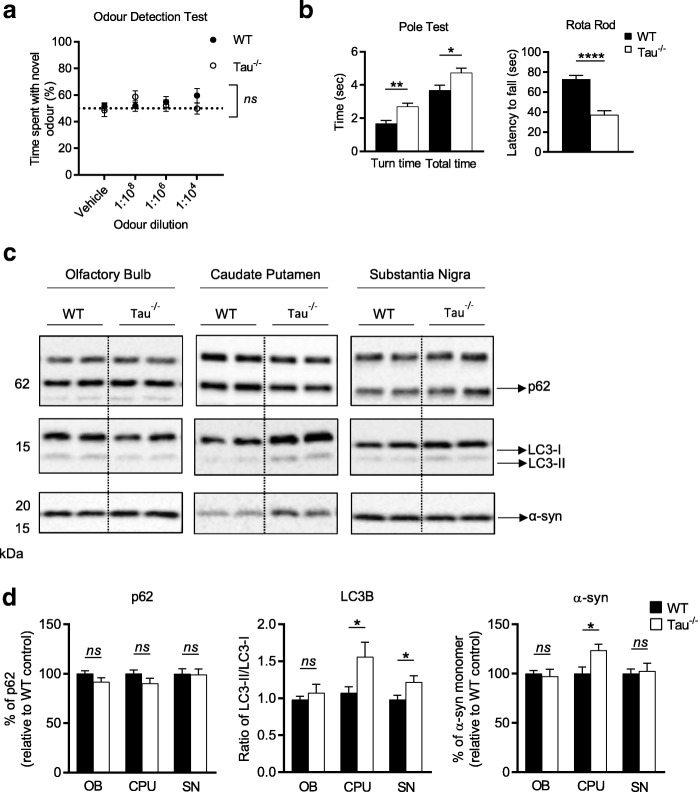
Olfactory deficit, motor performance and pathological features in 15-month-old tau^−/−^ mice. **a** Odour detection test performed on 15-month-old tau^−/−^ (*n* = 13) and littermate WT controls (*n* = 8). **b** Motor evaluation of 15-month-old tau^−/−^ (*n* = 11) and littermate WT control (*n* = 8), including Rota Rod and Pole Test performance. **c** Representative western blots of cell lysate from olfactory bulb, caudate putamen and substantia nigra from 15-month-old tau^−/−^ (*n* = 6) and littermate WT controls (*n* = 6) immunoblotted for p62, LC3B and α-syn. **d** Quantification of western blot densitometry presented as % of p62 relative to WT control, ratio of LC3-II/I relative to WT control and % of α-syn relative to WT control. Cell lysate for western blots normalised to automated total protein measurement via ChemiDoc stain-free detection software. ODT analysed by two-way repeated measures ANOVA (one factor repetition) with Fisher LSD post-hoc comparisons. Motor tests analysed by unpaired two-sided t test. Western blot analysed by unpaired two-sided t test from 3 independent repeats, * *p* < 0.05, ** *p* < 0.01, *** *p* < 0.001, **** *p* < 0.0001, *ns*: not significant. Full western blot images presented in Additional file 1: Figure S4

At 15 months old tau^−/−^ mice continued to display an odour detection deficiency, however WT mice were also displaying a hyposmic phenotype at this age, as demonstrated by a there being no main effect of genotype of *p* = 0.54 (Fig. 2a) when compared to tau^−/−^ mice and no significant difference to a hypothetical mean of 50% at any of the odour concentrations (Additional file 1: Table S3). The onset of motor impairment in these mice is reported to occur at 12 months, and is accompanied by dopaminergic neuronal degeneration in the SN and iron accumulation in dopaminergic neurons [41]. Based on this data we examined 12-month-old tau^−/−^ mice and found no significant difference in motor performance compared to WT controls, however there was still a robust olfactory deficit in tau^−/−^ mice (Additional file 1: Figure S1). As such, mice were further aged to 15-months-old and at this point tau^−/−^ performed significantly different on both the Rota Rod (*p* < 0.0001) and Pole Test (time to turn *p* = 0.004, total time *p* = 0.03) than WT controls (Fig. 2b).

Based on this behavioural deficit, we examined the autophagic markers and α-syn levels in the OB, CPU and SN of animals at 15-months-old. At this age the is no detectable difference in the OB between tau^−/−^ and WT mice (p62 *p* = 0.13, LC3-II/I *p* = 0.47, α-syn *p* = 0.73) (Fig. 2d). Although there was no difference in the level of p62 in either the CPU (*p* = 0.15) or the SN (*p* = 0.91), tau^−/−^ mice had a significant increase in the ratio of LC3-II/I in both brain regions (CPU *p* = 0.04, SN *p* = 0.03), indicative of impaired autophagy (Fig. 2d). In line with the early pathological features demonstrated in the OB of young animals, there was a congruent increase in α-syn in the CPU of older tau^−/−^ mice (*p* = 0.02) (Fig. 2d).

Overall these data suggest that in this animal model there are changes in protein levels that align with a behavioural phenotype in the olfactory system that eventually present in the midbrain alongside a motor deficit.

### Tau disruption and impaired autophagic clearance promotes the release of α-syn in association with exosomes

**Fig. 3:**
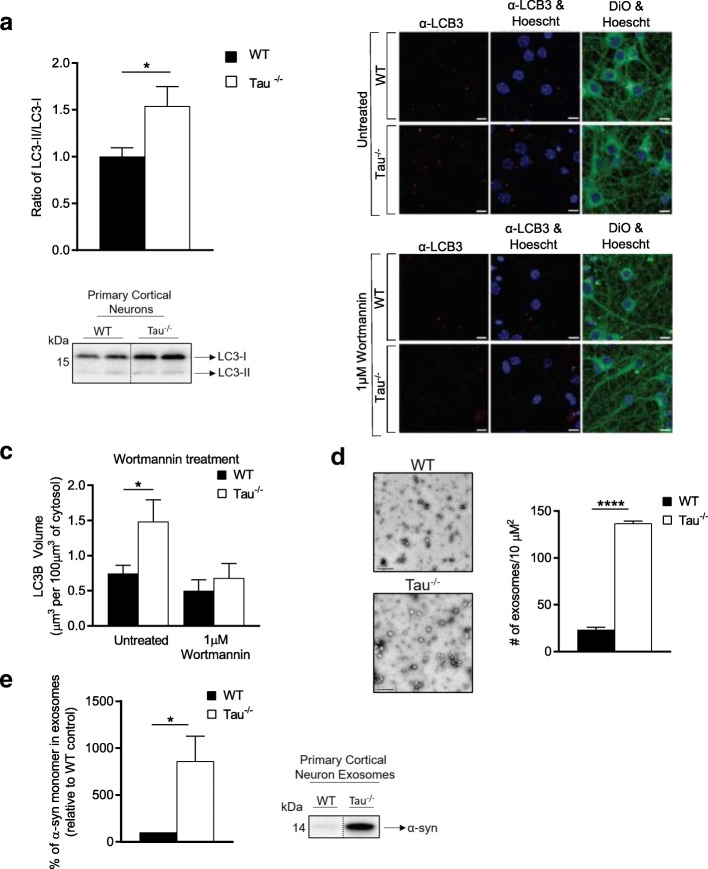
Tau^−/−^ primary cortical neurons display autophagic impairment and have an increase in the release of α-synuclein enriched exosomes. **a** Quantification of western blot densitometry presented as ratio of LC3-II/I of tau^−/−^ primary cortical neurons (*n* = 5) and WT primary cortical neurons (*n* = 5). **b** Representative images of tau^−/−^ and WT primary cortical neurons immunostained for LC3B and post-stained with DiO and Hoechst 33,342 after incubation with either culture medium (untreated) or 1 μM Wortmannin for 8 h, scale bars: 10 μm. **c** Quantification of the number of LCB3 positive autophagosomes per 100 μm^3^ of DiO stained cytosol, in untreated and Wortmannin treated WT (*n* = 5) and tau^−/−^ (*n* = 5) primary cortical neurons. **d** Representative electron micrograph images of tau^−/−^ (*n* = 3) and WT (*n* = 3) exosome enriched cell culture media (scale bars: 200 μm) with quantitation of number of exosomes per 10 μM^2^. **e** Quantification of western blot densitometry from exosomes isolated from tau^−/−^ (*n* = 2) and WT (*n* = 2) primary cortical neurons, presented as % of α-syn relative to WT control and representative western blot. Cell lysate for western blots normalised to automated total protein measurement via ChemiDoc stain-free detection software. Immunocytochemistry data analysed by one-way ANOVA. Western blot analysed by unpaired two-sided t test from 3 independent repeats, * *p* < 0.05, ** *p* < 0.01, *** *p* < 0.001, **** *p* < 0.0001, *ns*: not significant. Full western blot images presented in Additional file 1: Figure S5

To further investigate whether a loss of tau can cause an impairment in autophagy, we harvested primary cortical neurons from tau^−/−^ mice and their WT littermates and compared their autophagic flux. As we observed in the mouse tissue, there was a significant increase (p = 0.04) in the LC3-II/I ratio in the tau^−/−^ neurons as compared with WT neurons (Fig. 3a). Immunocytochemistry was also used to assess autophagosome number in WT and tau^−/−^ neurons (Fig. 3b). Quantitation of LC3B positive structures using semi-automated 3D image analysis revealed a significant increase in autophagosome number in tau^−/−^ neurons under basal conditions (*p* = 0.04) (Fig. 3c). Treatment with the autophagosome biogenesis inhibitor wortmannin resulted in no significant difference in the number of LC3B structures between WT and tau^−/−^, and an overall decrease of LC3B structures supporting that the structures represent autophagosomes. The data suggest that either there was a decrease in the lysosomal turnover of autophagosomes in tau^−/−^, or an increase in the amount of autophagy under basal conditions.

Impairment of autophagosome maturation or fusion with a lysosome has been suggested to result in fusion of autophagosomes to MVBs [64]. Once formed, MVBs can fuse with the plasma membrane to release their intraluminal vesicles as exosomes into the extracellular space. Exosomes are small vesicles released by cells and are major regulators of cell-to-cell communication in both pathological and normal conditions [57, 59]. Exosomes have been implicated in prion disease progression by promoting the cell-to-cell spread of pathological forms of the prion protein [23, 67]. The temporal displacement in the onset of disease relevant symptoms in the tau^−/−^ mice suggests a phenotype that is present at an earlier age in the OB spreads to the CPU/SN at an older age. Could this disease spread be facilitated through an exosome mediated mechanism? To test if loss of tau function could possibly contribute to exosome mediated disease spread we compared exosomes released into media from tau^−/−^ and WT mouse primary cortical cell cultures. Tau deletion resulted in an increase in the number of exosomes released into the media (*p* < 0.0001) (Fig. 1d) and an increase in the enrichment of α-syn within these exosomes (*p* = 0.049) (Fig. 1e). Isolated extracellular vesicles were verified as exosomes using a number of positive and negative exosome markers (Additional file 1: Figure S5C).

## Discussion

Variants of *MAPT* represent a risk factor in idiopathic PD and hyper-phosphorylation of tau is a consistent feature of many neurodegenerative diseases. Despite this evidence, the role tau plays in pathogenesis of PD has not been well characterised. Similarly, accumulation and aggregation of α-syn are histopathological hallmarks of PD brain and follow a defined pattern of spread with disease progression [10], however the mechanism(s) of protein aggregation and spread remain elusive. Our study demonstrates that a loss of the protein tau, via genetic knock out, resulted in a functional olfactory deficit coinciding with α-syn accumulation and autophagic impairments that began in the olfactory bulb and appeared in the midbrain 8 months later. These finding suggest that dysfunction of tau is an early pathological event in the neurodegenerative cascade associated with PD.

In this study, odour detection tests revealed an inability of 7-month-old tau^−/−^ mice to detect a novel odour. There have been numerous studies interrogating the tau^−/−^ mouse as potential model of PD [1, 16, 28, 41, 42, 48, 68]. Although there have been no published investigations into the olfactory capacity of tau^−/−^ mice to date, two tau-overexpressing models have reported olfactory dysfunction. Tα1-3RT tau transgenic mice that overexpress human tau and P301S tau mice that overexpress human mutant tau both demonstrate functional olfactory deficits [46, 72]. Although the exact cause of hyposmia has not been determined, there appeared to be a relationship between Lewy pathology and olfactory function [47, 51]. The delayed onset of motor impairment in this study (15-months-old) compared to published findings in this animal model (12-months-old) may be the result of genetic modifiers, as there have been differing results between models on different background strains in tau^−/−^ animals [42, 48]. The major motor and non-motor findings in the work by Lei et al. were reported in tau^−/−^ mice on the Sv129B/6 background strain. Shortly after this study, a number of groups were able to recapitulate the motor deficit in a pure black 6 (B6) background strain, however when it came to the non-motor phenotype (cognition), the B6 animals appeared to have either a delayed onset or no apparent phenotype [42, 48]. Based on this, it was deemed appropriate to use the background strain with demonstrated non-motor features. This background effect is one factor that may also explain a lack of olfactory capacity in WT controls at 15-months-old and requires further investigation.

Protein aggregates appear across multiple levels of the olfactory system [5] and both tau and α-syn are known to accumulate in neurodegenerative disease [10, 66]. Braak staging references an initial increase of α-syn in the anterior olfactory nucleus in human tissue, and in this study, there was an accumulation of α-syn in the olfactory bulb of hyposmic tau^−/−^ mice before midbrain pathological features appeared.

One explanation of the accumulation of α-syn in the tau^−/−^ mice may be disrupted clearance. The autophagy-lysosome pathway (ALP) is a fundamental mechanism used to remove misfolded and aggregated proteins [12, 55]. Defects in the ALP have been linked to PD [11, 52] and other neurodegenerative disorders (for review see: [70]). Increasing evidence suggests that dysregulation of autophagy results in the accumulation of abnormal proteins and/or damaged organelles, and impairments in the autophagy pathway have been implicated as a pathogenic feature of PD, as observed in PD brains as well as in animal models of PD [2, 7, 8]. Data from this study suggests there was impairment in autophagy in the olfactory bulb of 7-month-old tau^−/−^ mice, a deficit that appears in the midbrain in 15-month-old animals, correlating with motor deficits and α-syn accumulation in these brain regions. Although there is evidence of disrupted autophagic flux in 15-month-old animals, as demonstrated by an increase in the LC3II-I ratio, there was no notable change in p62 levels in the midbrain. While this was initially surprising, Sahani et al. have demonstrated in vivo that in distressed cells, there is an initial decrease in p62, but the cells were able to restore the level of p62 to basal levels [56]. Authors demonstrate that there are three major factors that control the expression level: autophagic degradation, transcriptional upregulation and availability of lysosomal-derived amino acids. Based on this, it is concluded that expression level of p62 does not always inversely correlate with autophagic activity and should only be taken in conjunction with other autophagic markers.

Many of the steps involved in autophagy require a functional cytoskeletal network. Disruption of dynein function leads to the accumulation of autophagosomes, suggesting that impairments to microtubule transport disrupt autophagosome-lysosome fusion [37, 71]. Tau is a microtubule-associated protein with a proposed role in microtubule stability; therefore, microtubule destabilisation following tau dysfunction is a potential mechanism of autophagy disruption. Data from this study demonstrates that ablation of tau in vivo lead to increases in the autophagy marker LC3B, suggestive of autophagy deficits that may lead to protein accumulation. It appears that although there is an increase in α-syn levels in the CPU at 15 months of age, the SN has no detectable difference between the WT and tau^−/−^ animals. Shimozawa et al. have investigated the distribution and spread of α-syn and they demonstrated a retrograde spread of pathology from the CPU to the SN [58]. It is possible that in this study the tissue has been collected at a time point that is reflective of the beginning of pathological pathways in the midbrain, reflected by the accumulation of α-syn only in the CPU. Autophagy deficits were confirmed in primary cortical neurons of tau^−/−^ neurons, supporting the principle that tau disruption results in altered autophagy.

α-syn aggregates are a histopathological hallmark of PD brain and follow a defined pattern of spread across the brain with disease progression. It was recently demonstrated that injection of exogenous α-syn into the brains of healthy animals could induce α-syn propagation in a “prion-like” manner and induce a PD phenotype [39, 44]. The specific mechanisms underlying the propagation of α-syn across the brain have however remained elusive.

Recent studies have shown that α-syn can be secreted via exosomes [4, 14, 20], although non-exosomal release of α-syn has also been described [14, 26]. Exosomes are the released form of intraluminal vesicles that are generated from invaginated membranes of multivesicular bodies (MVB) (reviewed in [9]). Exosome release is enhanced by impaired autophagosome-lysosome fusion, a key step in autophagy, as illustrated by genetic and pharmacological manipulations [19, 40]. The mechanism of this effect is not clear, although it has been proposed that autophagosomes may fuse with MVBs to subsequently effect fusion with the plasma membrane and release [14, 19, 22, 40]. Our study indicates that disruption of tau lead to autophagic impairment appearing initially in the olfactory bulb and later in the midbrain. Defective autophagy results in an accumulation of α-syn and in combination with autophagic impairments may drive the release of α-syn enriched exosomes, which may be an important mediator of α-syn spread.

## Conclusion

This study has demonstrated a link between tau ablation and autophagic disruption that coincides with α-syn accumulation and related behavioural deficits beginning in the olfactory system and eventuating in the midbrain. This implicates dysfunction of tau as an early pathological event in PD and signifies the value of tau^−/−^ mice as an age-dependent model of both prodromal and clinically overt Parkinson’s disease.

## Additional file

Additional file 1: Supplementary data **Table S1.** Animal numbers (genotype and sex). **Table S2.** ODT ANOVA factors. Normality test: Shapiro-Wilk. **Table S3.** ODT one sample t test, hypothetical mean = 50% (chance). **Figure S1.** ODT (A) and motor evaluation (B) of 12-month-old tau−/− (*n* = 12) and WT (*n* = 12). ODT analysed by two-way repeated measures ANOVA (one factor repetition) with Fisher LSD post-hoc comparisons. # represents significant main effect of genotype, ## *p* < 0.01. Motor tests analysed by unpaired two-sided t test. Ns = not significant. **Figure S2.** Western blot confirmation of tau ablation in 7- and 15-month old tissue. A) Tau antibody (Dako, catalogue number: A0024, dilution 1:10,000) confirmed tau ablation B) Protein loading in all wells confirmed by GAPDH (Cell Signaling Technology, catalogue number 2118, dilution 1:10,000). **Figure S3.** Full Western Blots of 7 mo WT (+) and Tau−/− (−) cell lysate. A, B, C: immunoblot of p62 from OB, CPU and SN respectively. D, E, F: immunoblot of LCB3 from OB, CPU and SN respectively. G, H, I: immunoblot of α-synuclein from OB, CPU and SN respectively. Red box indicates the section represented in Figures. **Figure S4.** Full Western Blots of 15 mo WT (+) and Tau−/− (−) cell lysate. A, B, C: immunoblot of p62 from OB, CPU and SN respectively. D, E, F: immunoblot of LCB3 from OB, CPU and SN respectively. G, H, I: immunoblot of α-synuclein from OB, CPU and SN respectively. Red box indicates the section represented in Figures. **Figure S5.** A) Full Western Blots of PCN for LC3B from WT (+) and Tau−/− (−) cell lysate. B) Full Western Blot of PCN-derived exosomes for α-synuclein from WT (+) and Tau−/− (−) cells. C) Exosome verification using exosome negative markers (GM130 & bcl-2), exosome positive marker (Tsg101) and microvesicle marker (Flotillin) in primary cortical neuron cell lysate and exosomes. (PDF 1104 kb)

## Abbreviations

ALP: Autophagy-lysosome pathway
BCA: Bicinchonic acid
BHT: Butylated hydroxytoluene
CPU: Caudate putamen
DTT: Dithiothreitol
ECL: Enhanced chemiluminescence
ITI: Inter-trial interval
Knockout: −/−
MVB: Multivesicular bodies
NFT: Neurofibrillary tangle
OB: Olfactory bulb
ODT: Odour detection test
PAR: Population attributable risk
PBS: Phosphate-buffered saline
PD: Parkinson’s disease
PFA: Paraformaldehyde
PTM: Post-translational modification
REM: Rapid eye movement
RIPA: Radioimmunoprecipitation assay
S.E.M: Standard error of the mean
SN: Substantia nigra
TBS: Tris-buffered saline
TEM: Transmission electron microscopy
WT: Wildtype
α-syn: Alpha synuclein
