# Supplementary material for: Ablation of tau causes an olfactory deficit in a murine model of Parkinson’s disease
Source: Acta Neuropathol Commun. 2018 Jul 5;6:57. doi: 10.1186/s40478-018-0560-y (PMC6032546; doi:10.1186/s40478-018-0560-y)
Supplement: Supplementary file 1 — Supplementary data Table S1. Animal numbers (genotype and sex). Table S2. ODT ANOVA factors. Normality test: Shapiro-Wilk. Table S3. ODT one sample t test, hypothetical mean = 50% (chance). Figure S1. ODT (A) and motor evaluation (B) of 12-month-old tau−/− (n = 12) and WT (n = 12). ODT analysed by two-way repeated measures ANOVA (one factor repetition) with Fisher LSD post-hoc comparisons. # represents significant main effect of genotype, ## p < 0.01. Motor tests analysed by unpaired two-sided t test. Ns = not significant. Figure S2. Western blot confirmation of tau ablation in 7- and 15-month old tissue. A) Tau antibody (Dako, catalogue number: A0024, dilution 1:10,000) confirmed tau ablation B) Protein loading in all wells confirmed by GAPDH (Cell Signaling Technology, catalogue number 2118, dilution 1:10,000). Figure S3. Full Western Blots of 7 mo WT (+) and Tau−/− (−) cell lysate. A, B, C: immunoblot of p62 from OB, CPU and SN respectively. D, E, F: immunoblot of LCB3 from OB, CPU and SN respectively. G, H, I: immunoblot of α-synuclein from OB, CPU and SN respectively. Red box indicates the section represented in Figures. Figure S4. Full Western Blots of 15 mo WT (+) and Tau−/− (−) cell lysate. A, B, C: immunoblot of p62 from OB, CPU and SN respectively. D, E, F: immunoblot of LCB3 from OB, CPU and SN respectively. G, H, I: immunoblot of α-synuclein from OB, CPU and SN respectively. Red box indicates the section represented in Figures. Figure S5. A) Full Western Blots of PCN for LC3B from WT (+) and Tau−/− (−) cell lysate. B) Full Western Blot of PCN-derived exosomes for α-synuclein from WT (+) and Tau−/− (−) cells. C) Exosome verification using exosome negative markers (GM130 & bcl-2), exosome positive marker (Tsg101) and microvesicle marker (Flotillin) in primary cortical neuron cell lysate and exosomes. (PDF 1104 kb) [file 40478_2018_560_MOESM1_ESM.pdf]

Additional file 1

	Sex	WT (n=)	Tau$^{-/-}$ (n=)
7 months old	*Male*	6	7
	Female	6	4
12 months old	*Male*	10	8
	Female	2	4
15 months old	*Male*	5	7
	Female	3	6

Table S1. Animal number breakdown based on genotype and sex.

Experimental Group	***Analysis***	**Parameter 1 Factor: genotype**	**Parameter 2 Factor: odour concentration**	**Parameter 3: interaction**
7 mo Sv129B/6	2-way RM ANOVA	$F_{1,57}$=11, p=0.003	$F_{3,57}$=5.3, p=0.003	$F_{1,57}$=2.2, p=0.01
12 mo Sv129B/6	2-way RM ANOVA	$F_{1,99}$=7.9, p=0.008	$F_{3,99}$=6.3, p<0.001	$F_{3,99}$=1.7, p=0.2
15 mo Sv129B/6	2-way RM ANOVA	$F_{1,66}$=0.39 p=0.54	$F_{3,66}$=0.55, p=0.65	$F_{3,66}$=1.01, p=0.4

Table S2. ODT ANOVA factors. Normality test: Shapiro-Wilk

Experimental Group	**Odour concentration**	**Hypothetical mean**	**Actual mean**	**SEM**	**n**	**p**
7 mo WT	0	50	49.46	3.67	11	0.88
	$1:10^8$	50	62.13	6.743	12	0.10
	$1:10^6$	50	68.39	3.672	10	0.0007
	$1:10^4$	50	72.89	3.613	10	0.0001
7 mo $tau^{-/-}$	0	50	45.04	2.53	10	0.08
	$1:10^8$	50	58.91	3.343	10	0.03
	$1:10^6$	50	46.79	5.355	11	0.56
	$1:10^4$	50	56.67	6.396	11	0.32
15 mo WT	0	50	51.40	2.785	8	0.63
	$1:10^8$	50	51.81	3.96	8	0.66
	$1:10^6$	50	54.76	4.06	7	0.29
	$1:10^4$	50	59.56	5.41	5	0.15
15 mo $tau^{-/-}$	0	50	48.78	5.27	12	0.82
	$1:10^8$	50	58.73	4.41	10	0.08
	$1:10^6$	50	52.15	4.27	12	0.63
	$1:10^4$	50	49.79	4.18	11	0.96

Table S3. ODT one sample t test, hypothetical mean = 50% (chance).

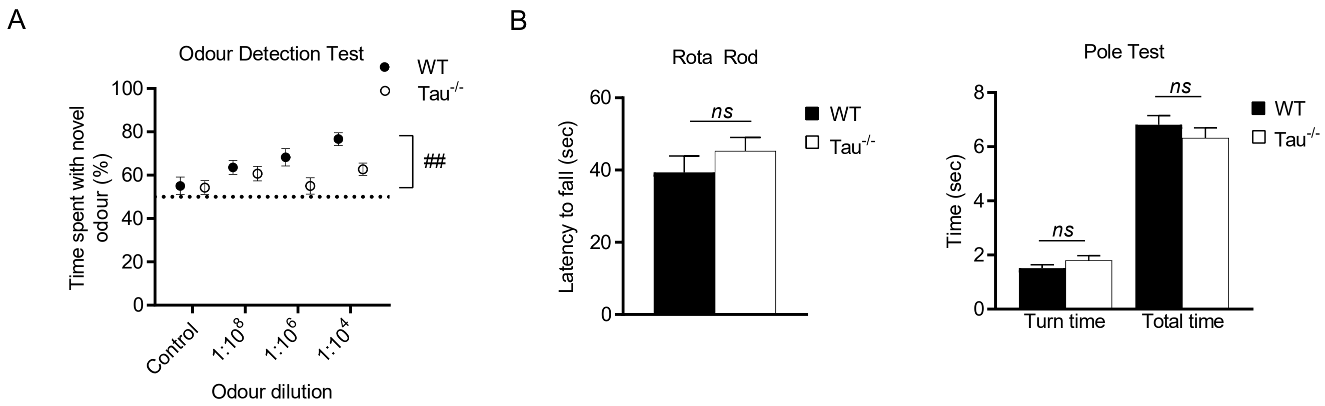

Figure S1. Odour detection test (A) and motor evaluation (B) (Rota Rod and Pole Test) of 12-month-old tau$^{-/-}$ (n=12) and littermate WT control (n=12). ODT analysed by two-way repeated measures ANOVA (one factor repetition) with Fisher LSD post-hoc comparisons. # represents significant main effect of genotype, ## $p < 0.01$. Motor tests analysed by unpaired two-sided t test. Ns = not significant.

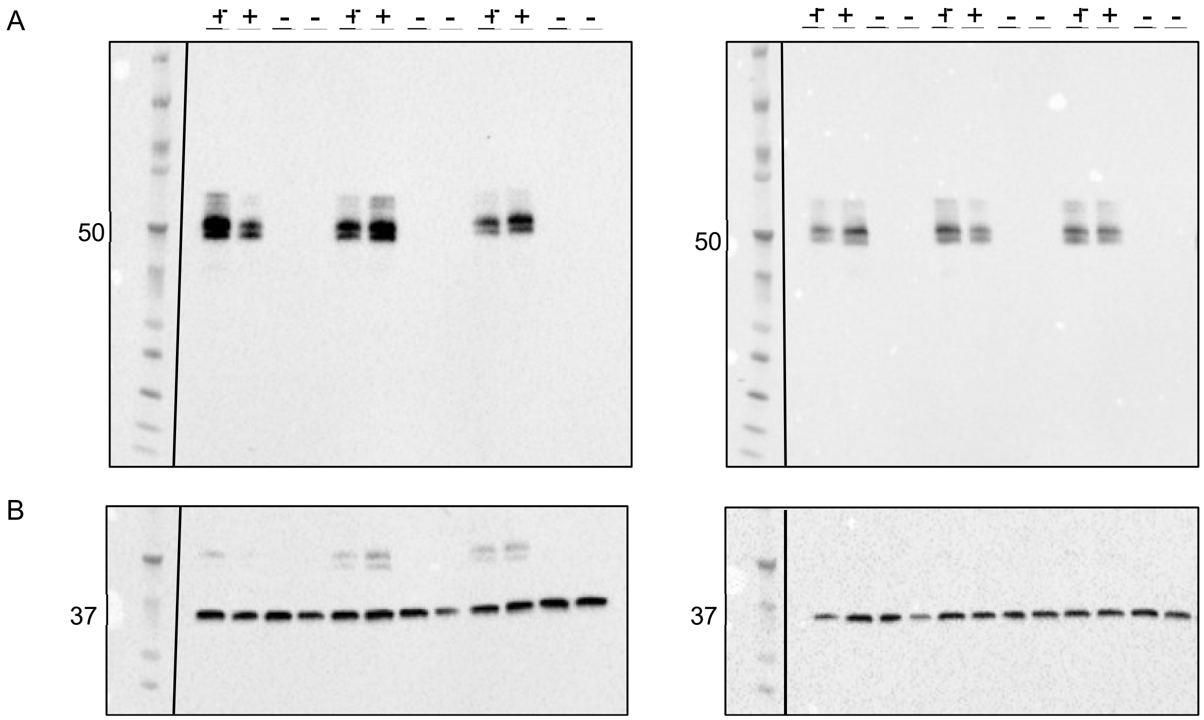

Figure S2. Western blot confirmation of tau ablation in 7- and 15-month old tissue used for immunoblot analysis. A) Tau antibody (Dako, catalogue number: A0024, dilution 1:10,000) confirmed tau ablation B) Protein loading in all wells confirmed by GAPDH (Cell Signaling Technology, catalogue number 2118, dilution 1:10,000).

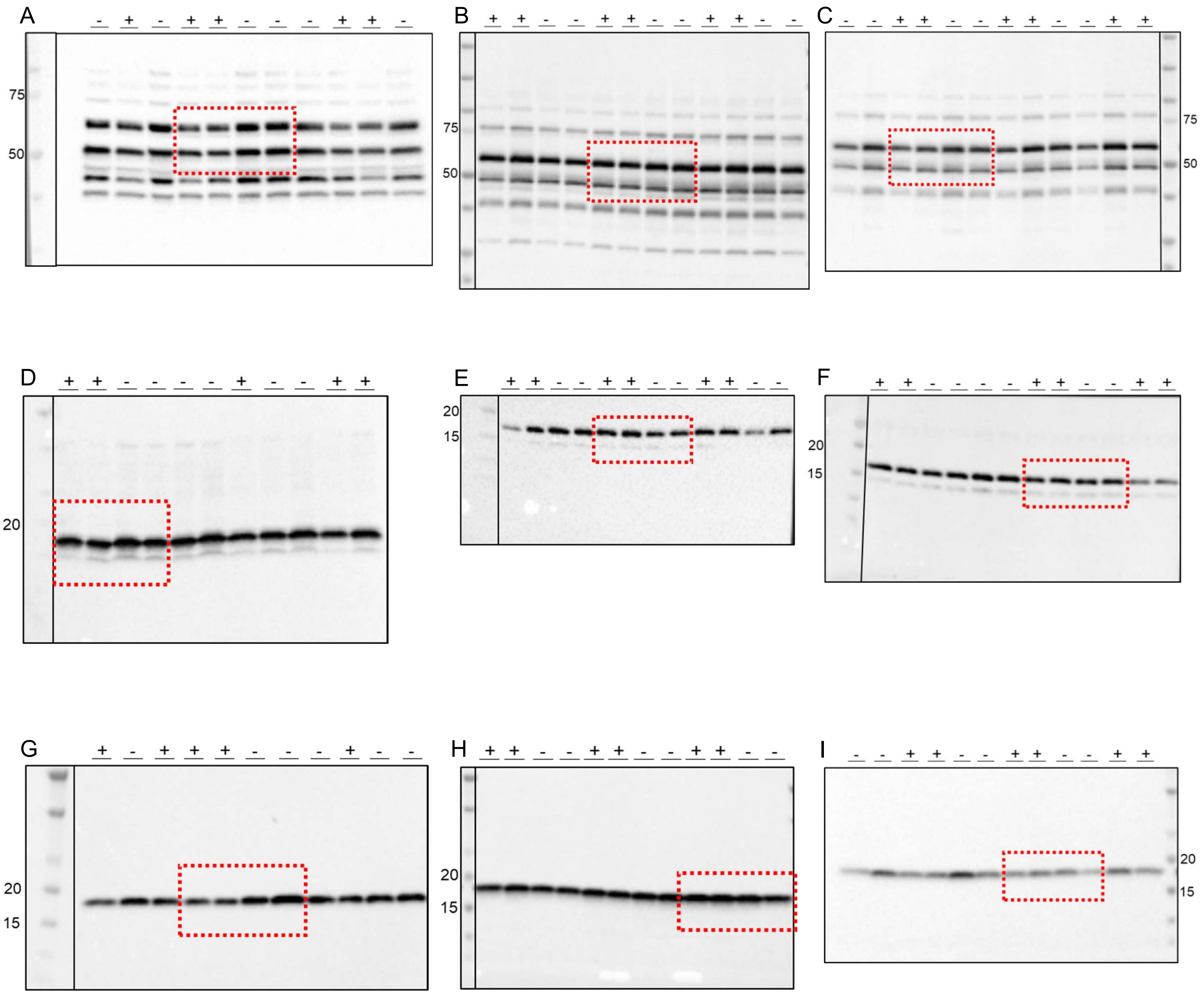

Figure S3. Full Western Blots of 7 mo WT (+) and Tau$^{-/-}$ (-) cell lysate. A, B, C: immunoblot of p62 from OB, CPU and SN respectively. D, E, F: immunoblot of LCB3 from OB, CPU and SN respectively. G, H, I: immunoblot of α-synuclein from OB, CPU and SN respectively. Red box indicates the section represented in Figures.

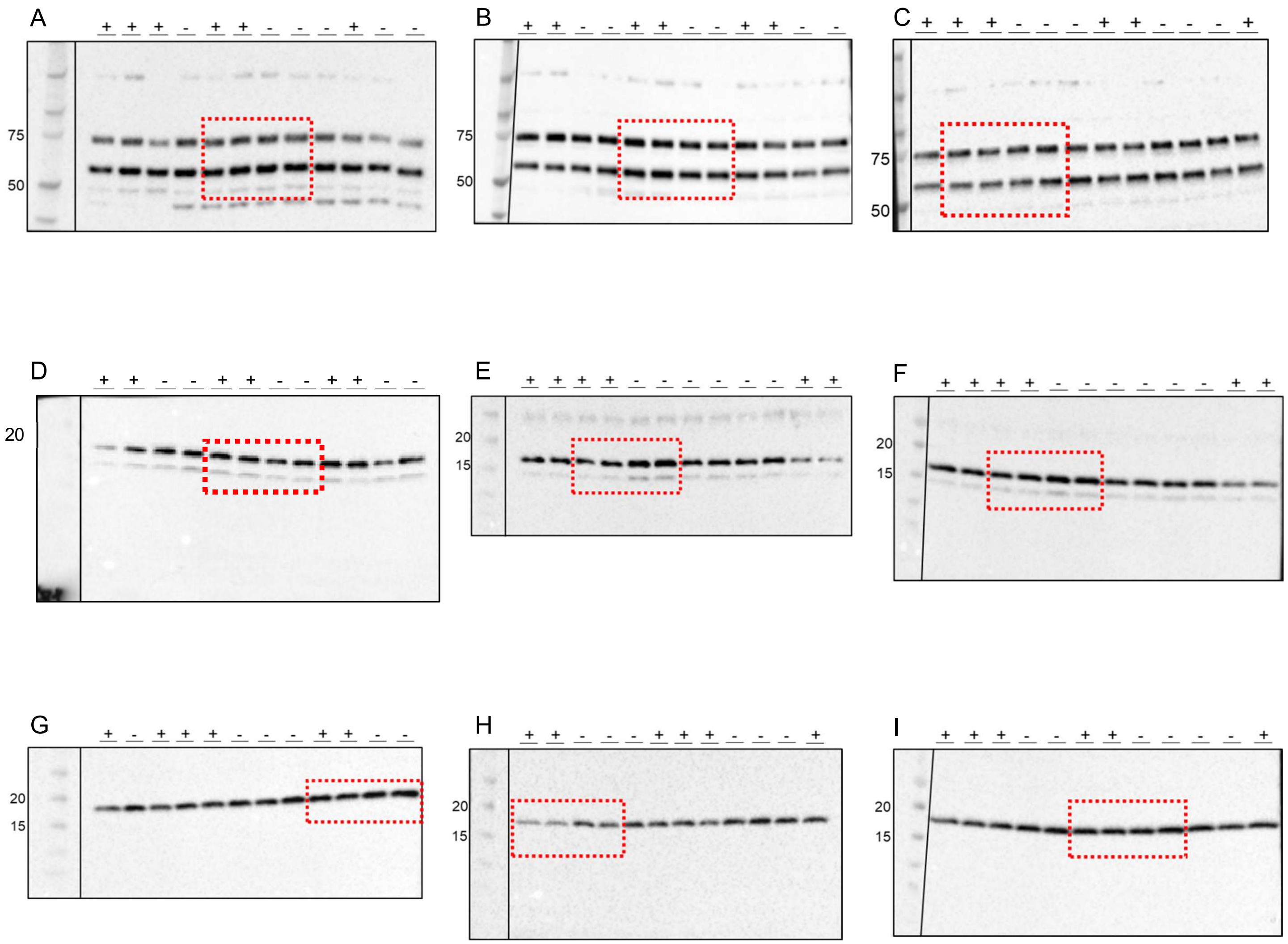

Figure S4. Full Western Blots of 15 mo WT (+) and Tau$^{-/-}$ (-) cell lysate. A, B, C: immunoblot of p62 from OB, CPU and SN respectively. D, E, F: immunoblot of LCB3 from OB, CPU and SN respectively. G, H, I: immunoblot of α-synuclein from OB, CPU and SN respectively. Red box indicates the section represented in Figures.

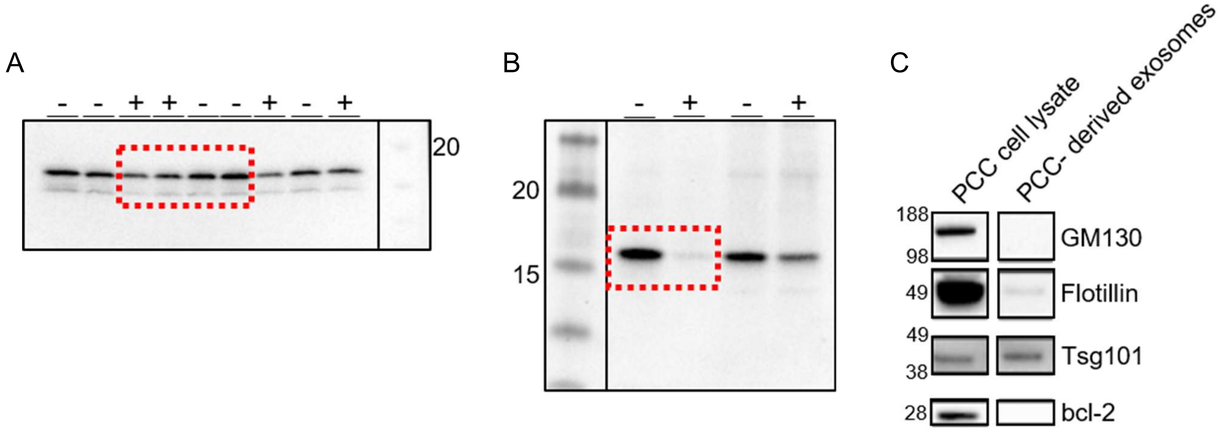

Figure S5. A) Full Western Blots of primary cortical neurons for LC3B from WT (+) and Tau$^{-/-}$ (-) cell lysate. B) Full Western Blot of primary cortical neuron-derived exosomes for α-synuclein from WT (+) and Tau$^{-/-}$ (-) cells. C) Exosome verification using exosome negative markers (GM130 & bcl-2), exosome positive marker (Tsg101) and microvesicle marker (Flotillin) in primary cortical neuron cell lysate and exosomes.
